# Characterization of Titanium Alloy Obtained by Powder Metallurgy

**DOI:** 10.3390/ma15062057

**Published:** 2022-03-10

**Authors:** Cristina Ileana Pascu, Claudiu Nicolicescu, Nicoleta Cioateră, Ștefan Gheorghe, Ionuț Geonea, Anca Didu

**Affiliations:** 1 Faculty of Mechanics, University of Craiova, 200512 Craiova, Romania; ileana.pascu@edu.ucv.ro (C.I.P.); stefan.gheorghe@edu.ucv.ro (Ș.G.); ionut.geonea@edu.ucv.ro (I.G.); 2 Faculty of Sciences, University of Craiova, 200585 Craiova, Romania; nicoleta.cioatera@edu.ucv.ro

**Keywords:** titanium alloy, two-step sintering (TSS), multiple-step sintering (MSS), crystalline structure, wear behavior, P/M composites, eco-friendly parts

## Abstract

Ti-based alloys are an important class of materials suitable especially for medical applications, but they are also used in the industrial sector. Due to their low tribological properties it is necessary to find optimal technologies and alloying elements in order to develop new alloys with improved properties. In this paper, a study on the influence of sintering treatments on the final properties of a titanium alloy is presented. The alloy of interest was obtained using the powders in following weight ratio: 80% wt Ti, 8% wt Mn, 3% wt Sn, 6% wt Aluminix123, 2% wt Zr and 1% wt graphite. Two sintering methods were used, namely two-step sintering (TSS) and multiple-step sintering (MSS), as alternatives to conventional sintering which uses a single sintering dwell time. Evolution of sample morphology, composition and crystalline structure with sintering method was evidenced. The lower values for the friction coefficient and for the wear rate was attained in the case of the sample obtained by TSS.

## 1. Introduction

Titanium has some spectacular properties, such as high toughness, low density and good corrosion resistance, making it attractive to be used most frequently in the biomedical field [1,2,3]. However, despite the very good physical and mechanical properties of titanium, the reason for its diminished area of applicability in the field of engineering is the high cost of titanium as a pure metal [4] and the difficult handling conditions, due to fine titanium powder flammability in contact with air [5] and low tribological properties [6]. For these reasons, titanium-based alloys are used in industrial applications [7], such as in the aerospace [8,9,10,11], automotive [12], chemical and petrochemical industries [13], as well as electronics and electrical engineering [14]. For automotive components, Ti-based alloys can be used as connecting rods, segments, part of the engine, chassis, pistons, valves, cranks and shafts [15,16,17,18].

Some researchers have found that the use of TiH_2_ powders can improve sintered density, induce microstructure modification, and reduce the oxygen content and cost of raw materials, compared with Ti powders [19]. Therefore, solutions were sought in the production of titanium alloys using cheaper manufacturing technologies from titanium compounds (TiO_2_, TiH_2_) [20].

However, the high friction coefficient and lower wear resistance than steel and Ni -based alloys have been a major drawback to its practical applications [21,22]. In this context, the addition of small quantities of metals, such as: Al, Sn, Mn and Fe, leads to improved wear resistance, while reducing manufacturing costs. The most used way to obtain titanium-based alloys at a lower cost is the PM technique [19,23,24,25]. The broad spectrum of PM processing routes provides advantages for the elaborated Ti-products: classical sintering, laser forming, powder injection molding, spraying, rapid solidification, mechanical alloying and vapor deposition [24,25,26,27].

Zr has chemical properties similar to Ti, and the formation of solid solutions causes the hardening of the alloy, increases the corrosion resistance and improves biocompatibility [20,28]. Although zirconium (Zr) and tin (Sn) are recognized as neutral elements, Zr and Sn can also improve the mechanical and corrosion properties when alloyed with Ti [29].

According to Lu et al. [30] the addition of a minor Mn element can remove Fe, being a good substitute for it, and improves the corrosion resistance and creep resistance of alloys; so, adding small percentages of Mn to a Ti-based alloy gives remarkable properties [31]. In [32], a novel near-α titanium alloy Ti-6.0Al-4.5Cr-1.5Mn was obtained by the water-cooled copper crucible and was observed to have better mechanical strength compared with other Ti-alloys but worse elongation.

The addition of aluminum in Ni-Ti alloy researched by Pavel Salvetr et al. showed the decrease of porosity and improvement of corrosion resistance and hardness [33]. Aluminum (Al) addition is due to its distinctive characteristics, such as low density (2.7 g/cm^3^) and combination of moderate strength and high ductility, thermal conductivity, electric conductivity, corrosion-resistance [34,35].

Previous studies concerning sintering procedure have shown that two-step (TSS) and multiple-step (MSS) sintering techniques allow researchers to obtain homogeneous compositions and microstructures of Ti-based alloys at low cost [36]. The processing of titanium alloys is increasing in the industry, since these alloys present superior mechanical properties to commercially pure titanium [37].

The selection of the processing methods is a critical factor that influences the microstructure, phase formation and its kinetics, as well as mechanical and tribological properties of any material. Therefore, the final properties and suitability to different applications can be tailored by fine-tuning of a selected production technique [38]. Previous research [39] has revealed that the sintering of titanium at temperatures between 1000–1150 °C did not lead to an accentuated densification of the final alloy nor any remarkable differences of its final physical-mechanical properties. The main reason was considered to be the relatively short dwell time on the sintering temperature.

Therefore, in order to optimize the sintering process of the titanium-based alloy, it was considered of interest to use the sintering temperature of 1050 °C, with the increase of the dwell time to 100 min. At the same time, in order to find more economical solutions in terms of energy and the total cost of obtaining the titanium-based alloy, the two types of treatments presented in this paper were chosen: TSS and MSS.

The stated goal of this research is to find cheaper technologies for obtaining titanium-based alloy, in order to eliminate the main obstacle in the use of titanium, especially in the automotive field, namely the high cost of pure titanium. Therefore, it acted in two directions: (i) the use of titanium hydride as a raw material and (ii) the use of cheaper but more modern technologies, compared to classical sintering.

Given the previous results obtained by adding small amounts of metallic elements that allowed to obtain Ti-based alloys with improved final properties, we developed a Ti-based alloy with the following chemical composition: 80% wt. TiH_2_, 8% wt. Mn, 3% wt. Sn, 6% wt. Alumix321, 2% wt. Zr and 1% wt. graphite. This Ti-based alloy was obtained by PM and was subjected to two types of sintering processes: TSS and MSS. The main purpose of this work was to identify the most appropriate sintering procedure which allows the decrease of the manufacturing cost and the optimal properties for industrial applications, respectively, for components in the automotive industry.

## 2. Materials and Experimental Methods

### 2.1. Material Selection

The following raw materials were used in the production procedure:

- Micronic hydride powder (TiH_2_) with particle size min. 99.9% <63 µm, water atomized produced by Chemetall GmbH (Frankfurt, Germany), as a basic metal matrix, having titanium min. 95%, hydrogen min. 3.8%, small amounts of Si, Mg, Ni, Al, Fe max. 0.1% for each of them;
- Micronic manganese powder, type MN006020, Goodfellow company (Seoul, Korea) provenience, purity 99.5% used for improving the wear behavior in friction conditions due to its property to form a protective layer of oxide in contact with air [40];
- Tin micronic powder, SN006020, produced by Goodfellow company (Tokyo, Japan); tin helps titanium in the grinding process and is a metal with low influence in the transformation temperature; it also behaves very well in friction conditions [41];
- Zirconium nanometric powder, purchased from the Goodfellow company (Hamburg, Germany). Zirconium has the same atomic structure as titanium, being considered an excellent alloying element of titanium, with a good corrosion resistance and has influence on reducing the transformation temperature [36,42];
- Graphite powder, 2N5 type from American Elements company (Los Angeles, California, USA), having 99.5% purity and low friction coefficient. Due to Van der Waals forces, graphite has a very good wear resistance, being an excellent dry lubricant, these being the reasons why it was added in the composition of the alloy [43];
- Alumix powder Al 123, based on aluminum powder but with small amounts of 4.5% wt. Cu, 1.31% wt. Mg, 0.5% wt. Si, 0.10% Fe, 0.05% wt. Sn 2%.

### 2.2. Experimental Procedure

The selected powders were blended and mechanically alloyed under Ar atmosphere for 120 min using a planetary mono mill PULVERISETTE 6 from Fritsch, (Idar-Oberstein, Germany) (grinding balls and bowl of silicon nitride). The weight ratio between the components were: 80%wt. TiH_2_, 8%wt. Mn, 3%wt. Sn, 6%wt. Alumix321, 2%wt. Zr and 1%wt. graphite.

In order to obtain the green parts (not sintered), two grams of mixture were weighted using a Partner WPS 510/C/2 (Bucharest, Romania) balance, followed by the uniaxial pressing process with a 600 MPa pressure, at room temperature and without lubrication. For the compaction process, a X200Cr120 cylindrical die was used.

**Figure 1:**
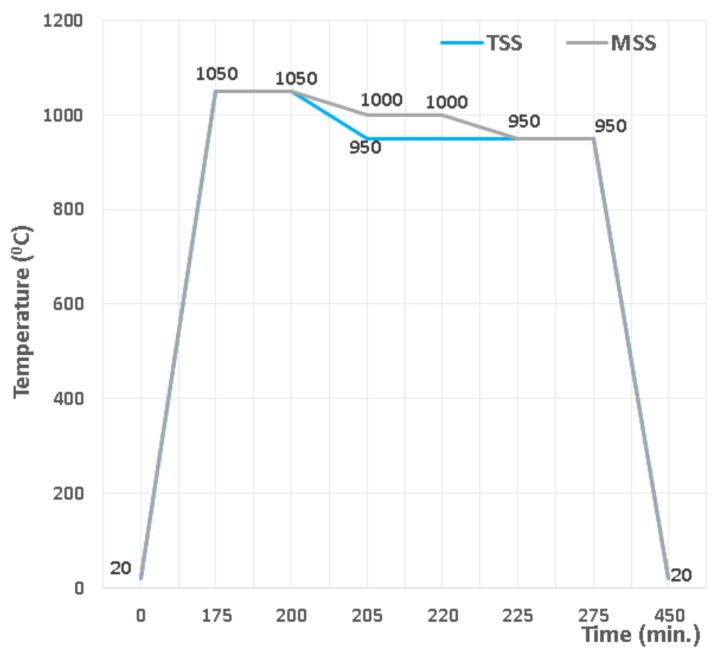
Sintering processes flow chart.

For the green compact’s densification, two sintering treatments were chosen, as shown in Figure 1.

According to Figure 1, the TSS and MSS processes consist of:

- sintering in two steps (TSS), with heating up to 1050 °C as the first stage, dwell time of 25 min and then the second sintering stage at 950 °C, with a dwell time of 75 min.
- sintering in multiple steps (MSS), the first sintering level being at 1050 °C, with a dwell time of 25 min, the second sintering level at 1000 °C, with a dwell time of 20 min and the third sintering level at 950 °C, with a dwell time of 55 min.

The heating rate was 6 °C/min for both sintering types. The green compacts were heated into a Nabertherm L3/11/C6 (Bucharest, Romania) furnace in Ar atmosphere.

After sintering, the samples were cooled down to room temperature in the furnace.

### 2.3. Characterization Techniques

The morphology and elemental analysis of the precursor powders and sinters were evidenced using a scanning electron microscope (SEM) from Hitachi (SU 8010, Mannheim, Germany) coupled with energy dispersive X-ray spectroscopy (EDXS) (Oxford Instruments, Mannheim, Germany). For this purpose, the TSS and MSS specimens were axial sectioned using a Metkon Metacut-M 250 (Bucharest, Romania) cut-off machine and heat-mounted in transparent resin, using a Metkon Metapress-A (Bucharest, Romania) mounting press. After that, the samples were grinded with silicon carbide papers with the following grits: 80, 120, 240, 400, 800, 1200 and 2000. Finally, the samples were polished with a GALAXY Polishing cloth PHI (Standard Service, Bucharest, Romania) and Dia-Duo 2 suspension (Struers, Bucharest, Romania). For grinding and polishing processes, a Metkon Forcipol 2V (Bucharest, Romania) machine was used.

In order to study the particle size distribution of powders, the dynamic laser scattering (DLS) technique was used, and the equipment was a Brookhaven 90Plus by Brookhaven Instruments Corporation (Wien, Austria). The samples were dispersed in water and subjected to the ultrasonication for 5 min.

X-ray diffraction (XRD) was used to perform the crystalline phase analysis in the sintered bodies (SmartLab from Rigaku, Neu-Isenburg, Germany).

Tribological tests were performed with a TRB 01-2541 tribometer, using TriboX software (version 2.10, Peseux, Switzerland), while a Surtronic S25 Taylor Hobson Profilometer (Leicester, UK) was used to measure the worn track section. In these cases, a steel ball 440C was used as counterpart and titanium-based alloy samples with a diameter of 12 mm obtained by TSS and MSS were used as tested parts. The testing conditions were: testing type—linear, normal load—2N, amplitude—6 mm, testing distance—24 m, atmosphere—air.

After the friction coefficient measurements, the worn track sections of the samples and the balls used were analyzed using a Nikon MA 100 microscope (Amsterdam, The Netherlands) equipped with NIS ELEMENTS software (Documentation version, Amsterdam, The Netherlands).

In order to calculate the wear rates, the worn track sections were measured using the Surtronic 25+ profilometer (Leicester, UK), and the formula used is given by:

(1) $$W=\frac{V_{\mathrm{loss}}}{F\cdot D}$$

where V_loss_ is the volumetric loss of the sample, F is the force applied and D is the total sliding distance.

## 3. Experimental Results and Discussions

### 3.1. Precursor Powder Characterization

**Figure 2:**
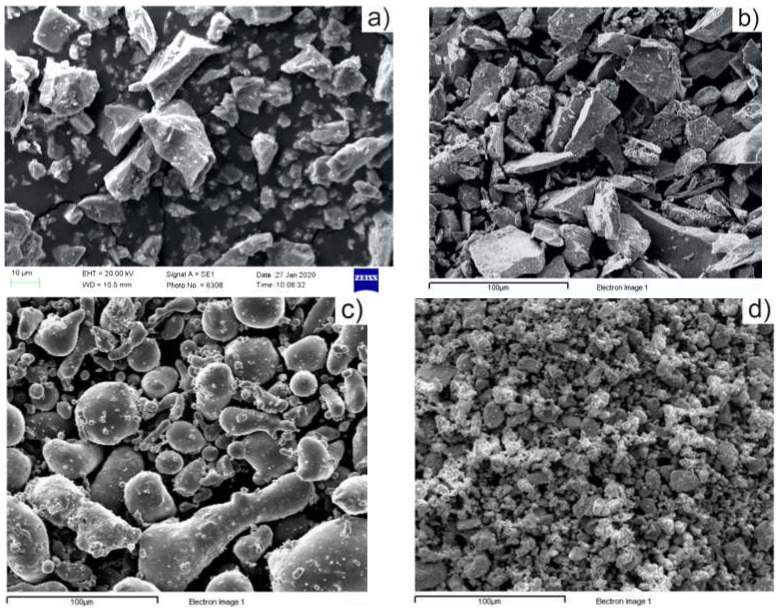
Scanning electron microscopy (SEM) images of: (**a**) TiH_2_ powder, (**b**) Mn powder; (**c**) Sn powder; (**d**) Zr powder.

**Figure 3:**
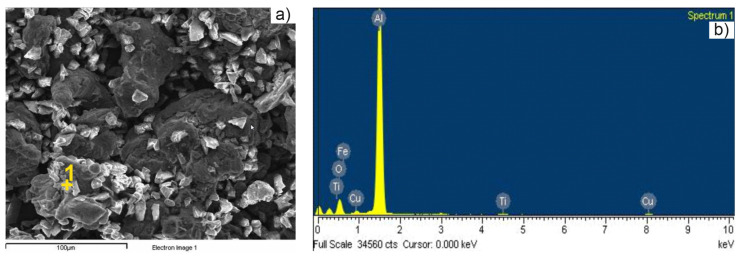
Alumix powders: (**a**) SEM image and (**b**) energy-dispersive X-ray spectroscopy (EDXS) spectrum.

SEM images of precursor powders are shown in Figure 2a–d. Particles with irregular and angular geometry that embody a great benefit during compaction step can be observed for TiH_2_ and Mn powders (Figure 2a,b), while round-shape particles were evidenced in Sn and Zr powders (Figure 2c,d). SEM image and EDXS spectrum of Alumix powder are shown in Figure 3.

The hydrodynamic diameter of precursor mixture measured by DLS can be slightly larger compared to that measured at SEM because DLS measures the diameter of a sphere, which has the same average diffusion coefficient as the measured particle [44].

**Figure 4:**
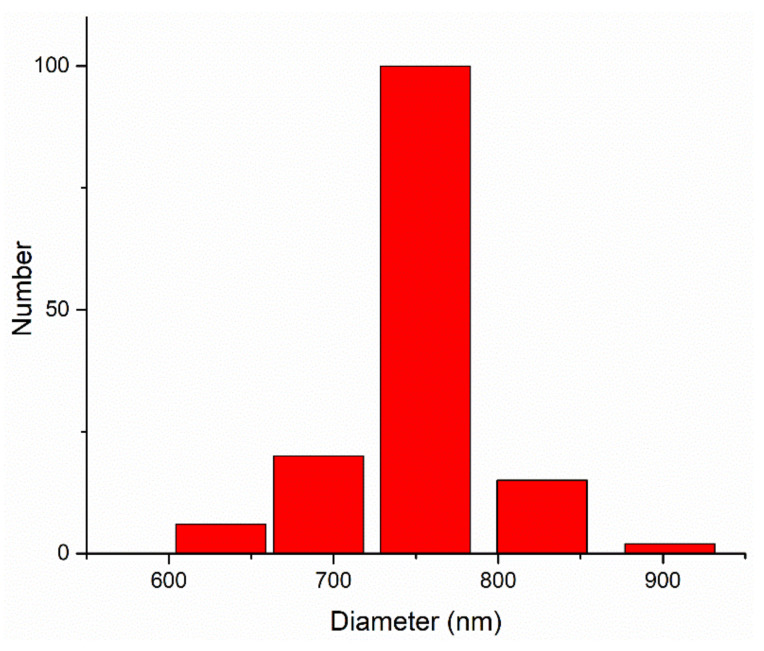
Numerical particle size distribution of the mixture.

In Figure 4 and Table 1, the numerical particle size distribution of the mixture is shown.

As it can be seen from Figure 4 and Table 1, the particle size distribution is in the range of 631–904 nm with the following structure: 4% of the total number of particles are around 631 nm, 14% of the total number of particles are around 690 nm, 70% of the total number of particles are around 755 nm, 10% of the total number of particles are around 826 nm and 2% of the total number of particles are around 904 nm.

**Figure 5:**
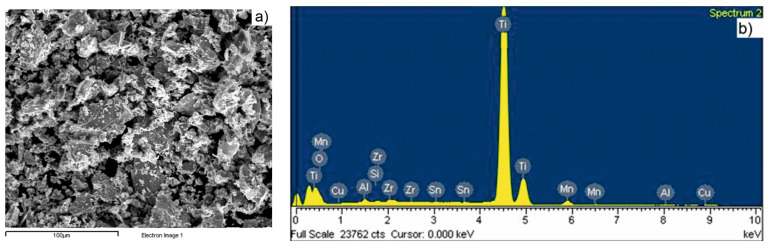
The mixture based on TiH_2_ powder: (**a**) SEM image and (**b**) EDXS spectrum.

The morphology of the mixture, as green pellet (after die compaction), is shown in Figure 5a, and the EDXS analysis is presented in Figure 5b.

**Figure 6:**
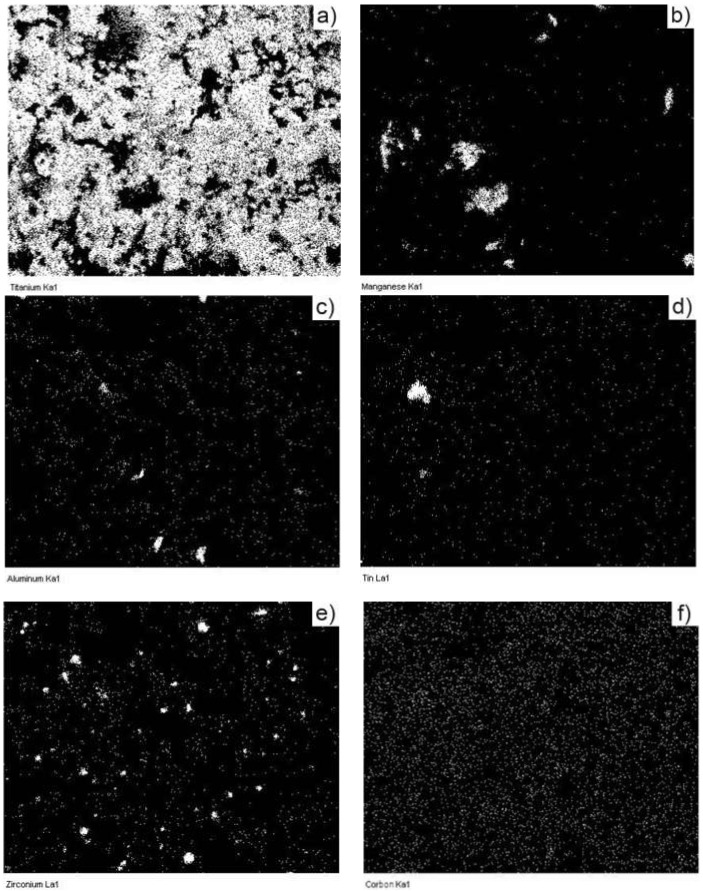
Elemental mapping evidencing the component’s dispersion in the mixture: (**a**) Ti; (**b**) Mn; (**c**) Al; (**d**) Sn; (**e**) Zr and (**f**) Cu.

In Figure 6, the dispersion mode of each component of the mixture is shown using elemental mapping of green compact. The massive dispersion of titanium powder can be observed, while Cu powder is uniformly dispersed in the mass of the mixture. However, small agglomerations appear for Mn and Zr powders.

### 3.2. Sintered Pellet Characterization

**Figure 7:**
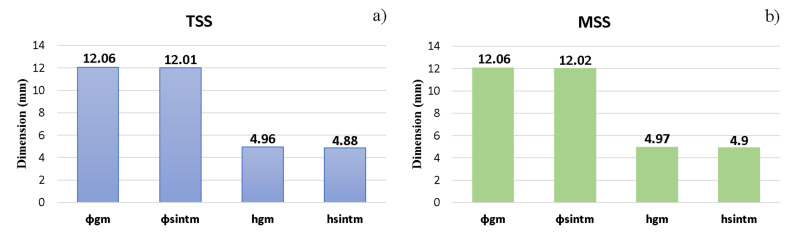

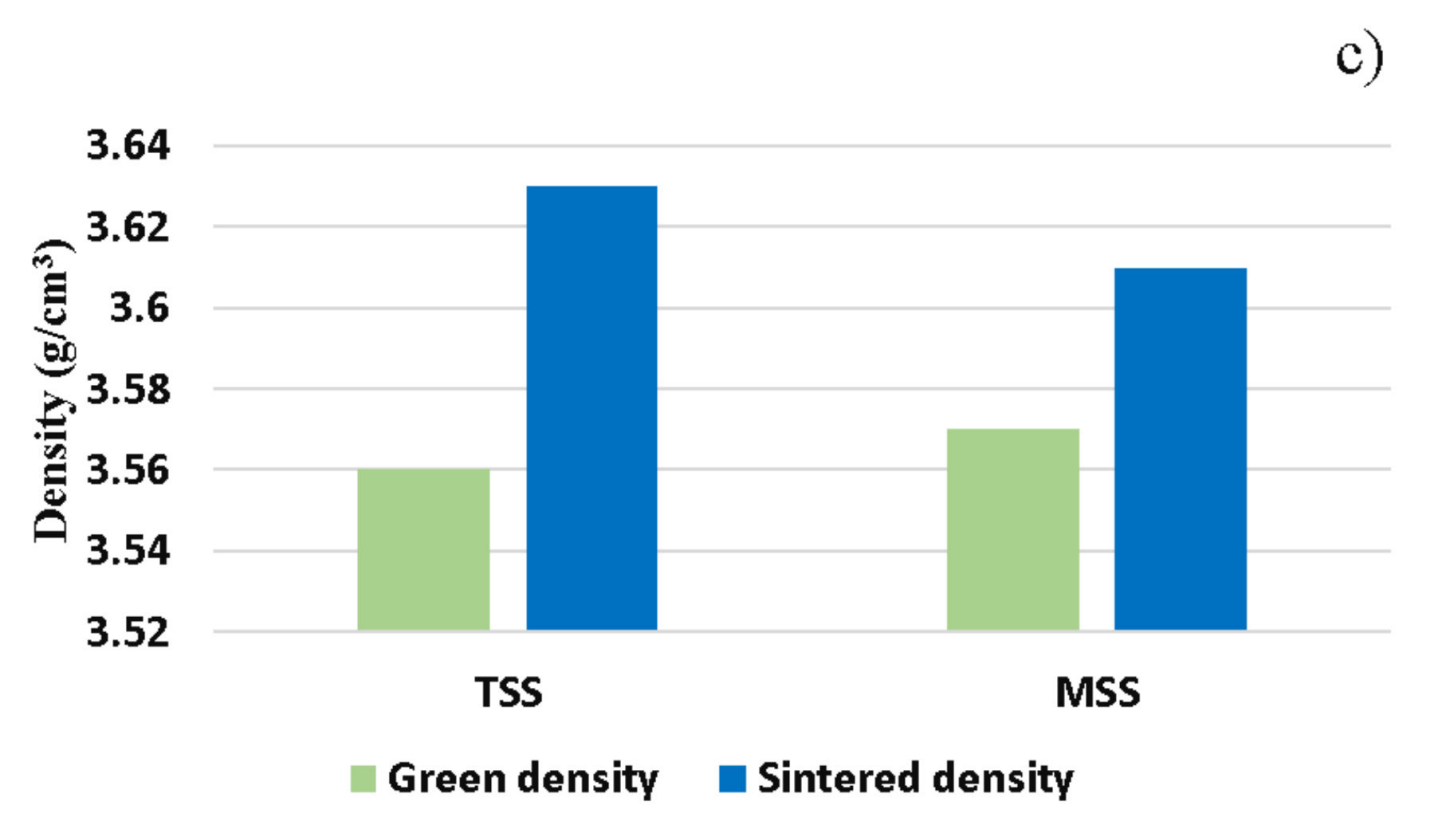
The final physical properties: (**a**) the average dimensions for green and sintering samples, obtained by two-step sintering (TSS); (**b**) the average dimensions for green and sintering samples, obtained by multiple-step sintering MSS; (**c**) green and final density versus sintering cycle (ϕgm—the diameter measured after compaction, ϕsintm—the diameter measured after sintering, hgm—the height of the parts after pressing and hsintm—the height of the parts measured after sintering).

During the sintering process, the TiH_2_ precursor powder has suffered a dehydrogenation process leading to Ti-based alloy. After sintering, the variations in sample height and diameter during shrinkage were measured. In Figure 7, the graphical variations in average values of Ti-based sample heights and diameters for the TSS (Figure 7a) and MSS (Figure 7b) cycles are shown.

Figure 7a,b show that the shrinkage in the height and diameter of the specimens present a downside slope. This behavior confirms the densification process of the Ti-based alloys.

After compaction and sintering, the green and the final density of the parts have been established by measuring their dimensional sizes and weighing them with an analytical balance, and then, the density formula for cylindrical parts has been applied. In Figure 7c, a comparative analysis between the values obtained after compaction and sintering density for each sintering cycle is given

**Figure 8:**
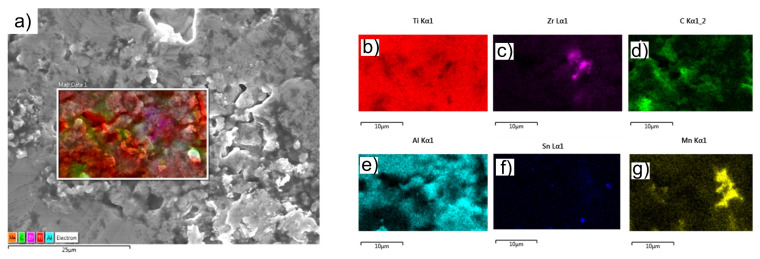
SEM image and EDXS elemental mapping for the sample obtained by TSS: (**a**) SEM image and overlayed maps, (**b**) Ti, (**c**) Zr, (**d**) C, (**e**) Al, (**f**) Sn and (**g**) Mn map distributions.

In Figure 8, the SEM microstructure and EDXS elemental mapping for the sample obtained by TSS is shown.

Figure 8 shows a relatively homogeneous distribution of Al in Ti matrix, while Zr, Mn and Sn formed islands in Ti matrix. TiH_2_ dehydrogenation during the sintering treatment is indicated by the presence of pure titanium grains in high ratio (over 87%; see Table 2). The high carbon content at the surface is due to the exposure of the surface layer of the parts to the environment when simultaneous oxidation and carburization of the surface layer occur [45,46,47].

**Figure 9:**
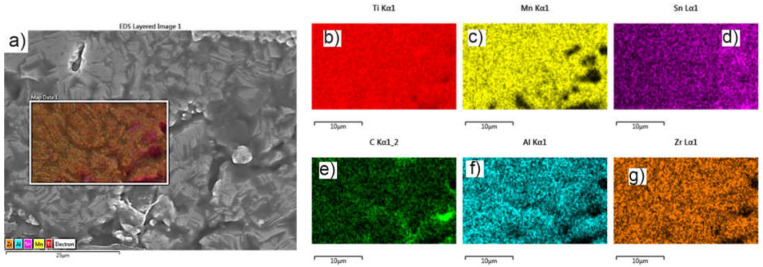
SEM image and EDXS elemental mapping for the sample obtained by MSS: (**a**) SEM image and overlayed maps, (**b**) Ti, (**c**) Mn, (**d**) Sn, (**e**) C, (**f**) Al and (**g**) Zr map distributions.

In Figure 9, the SEM microstructure for the sample obtained by MSS is shown. The sample exhibits a homogeneous distribution of all the alloying elements in the sample. However, the porosity of the sample obtained by MSS seems to be higher compared to those obtained by the TSS process, in agreement with density measurements.

**Figure 10:**
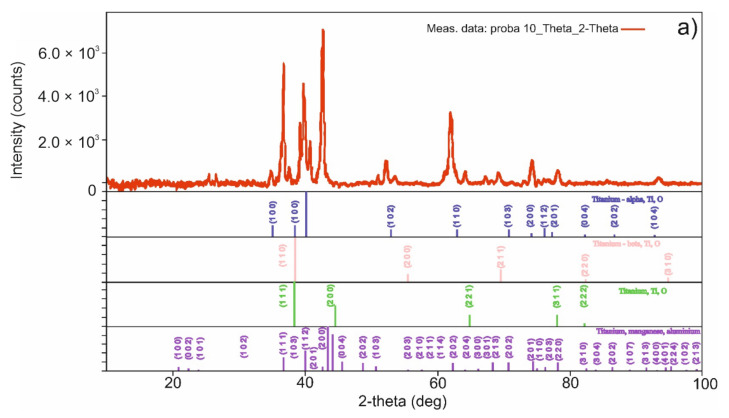

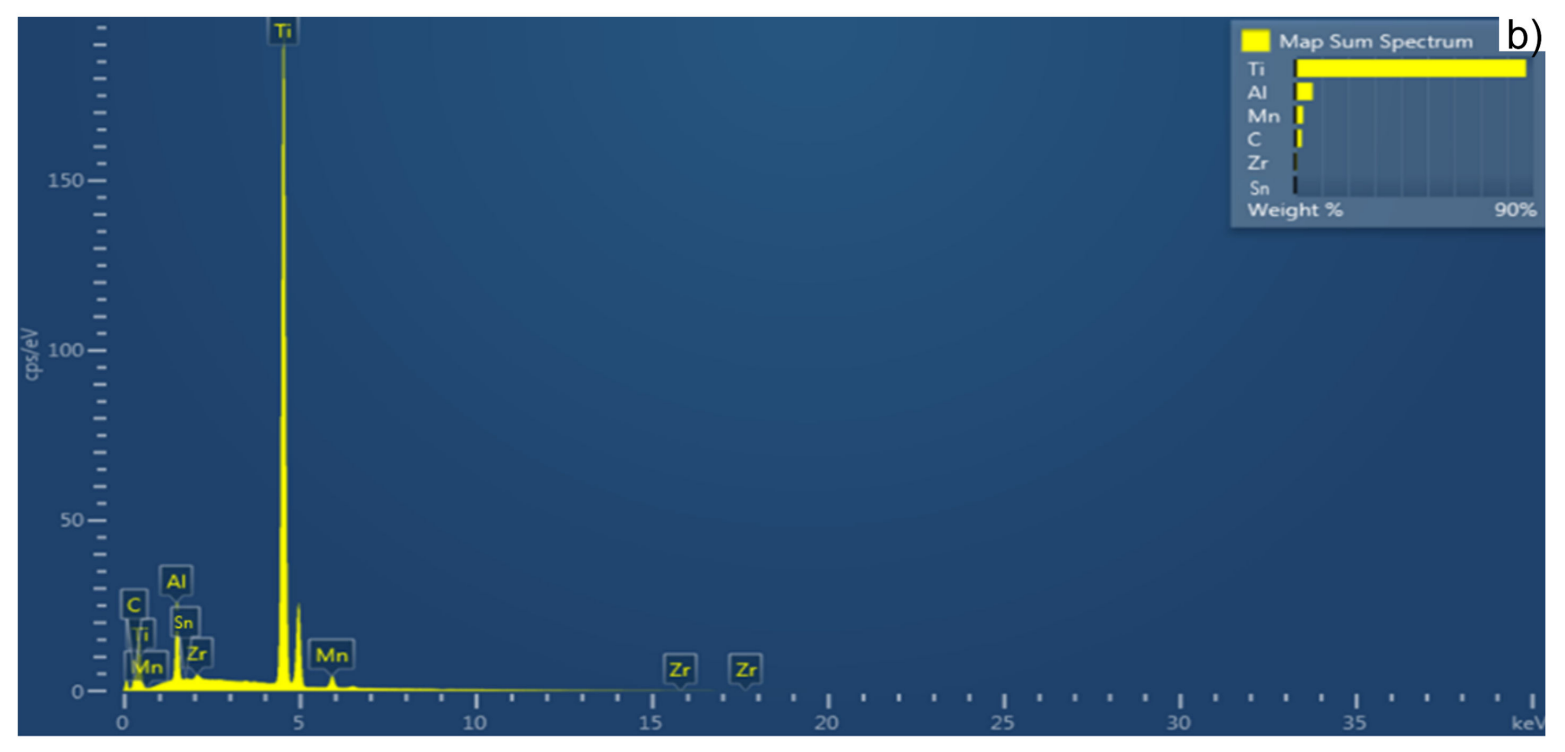
The X-ray diffraction (XRD) (**a**) and energy dispersive X-ray analysis EDX (**b**) for sample obtained by TSS.

In Figure 10, the X-ray diffraction pattern (Figure 10a) and energy dispersive X-ray analysis (EDX) (Figure 10b) of the sample obtained by TSS are presented.

**Figure 11:**
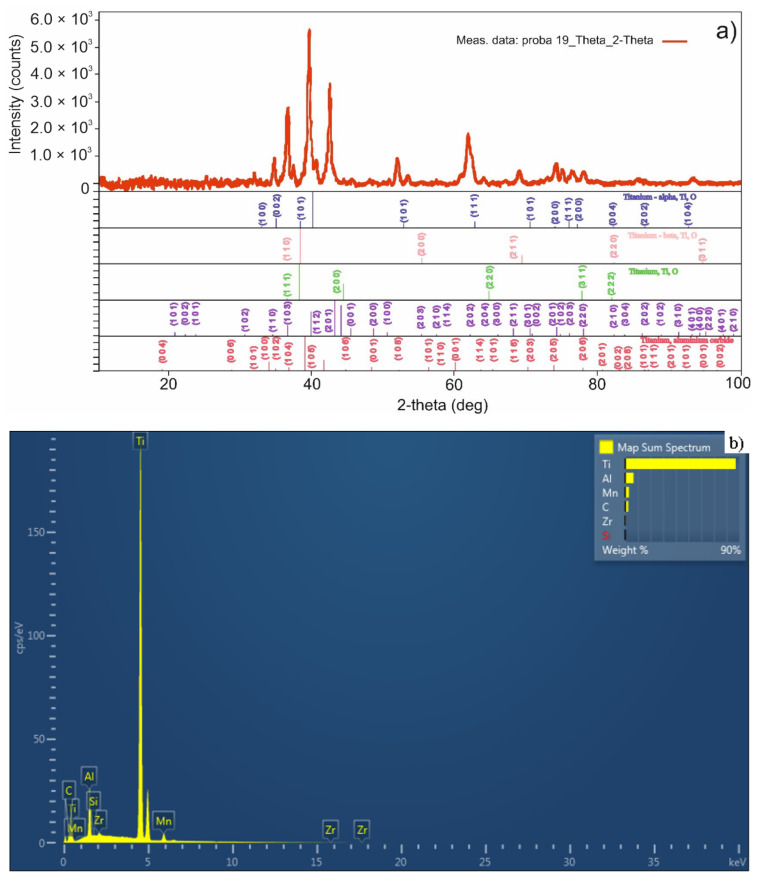
The X-ray diffraction (XRD) (**a**) and EDXS (**b**) for sample obtained by MSS.

For the sample obtained by MSS, in the Figure 11a, the X-ray diffraction (XRD) pattern and in the Figure 11b, the energy-dispersive X-ray (EDX) spectrum are shown.

The results of EDX analysis are presented in Table 2. It is worth mentioning that elements with atomic numbers higher than Be were detected using our Oxford Instrument detector, and titanium was the major component in both samples.

The EDX analysis asserts the tendency of agglomeration for the alloying elements in MSS sample and an increase of the carbon content due to the oxidation of the surface layer and the appearance of carburizing and nitriding phenomena. The presence of a low amount of Si is due to the contamination of the powder with the material of the grinding bowl and balls (silicon nitride).

Using XRD analysis, crystalline phases present in the investigated samples were evidenced. Thus, in the sample obtained by TSS (Figure 10a) three crystalline phases ascribed to different titanium polymorphs were identified: cubic (space group F m—3 m), titanium beta with cubic symmetry (space group I m—3 m), titanium alpha with hexagonal symmetry (space group P 63/mmc). In addition to those, the characteristic reflexions of an intermetallic compound were evidenced (Ti:Mn:Al = 1/1.56/0.44, space group P 63/mmc, with hexagonal symmetry). The as-prepared sample showed a similar XRD pattern with the sample obtained in a single sintering step at 1100 °C [47]. We can conclude that the decrease in thermal treatment temperature from 1100 °C to 1050 °C and the addition of a second step at 950 °C lead to the same crystalline phases in the sinters. However, a decrease in the intensity of the characteristic peaks was evidenced, indicating a decrease in crystallite sizes as was expected. For the sample obtained by MSS (Figure 11a), a supplementary crystalline phase was identified beside the ones from TSS sample, namely titanium aluminum carbide (3/1/2) with hexagonal symmetry and space group P 63/mmc.

### 3.3. Tribological Behavior of Titanium Based Alloy

In Table 3, the specific characteristics for the conducted wear tests for each sample are shown.

As Table 3 shows, the value of the friction coefficient of the TSS sample (µ = 0.545) is lower than the friction coefficient of the MSS sample (µ = 0.567), and this is probably due to the higher density of the TSS sample (3.63 g/cm^3^) than the density of the MSS sample (3.61 g/cm^3^).

The average of the penetration depth in the case of the sample obtained by the TSS method is −9.596 × 10^−3^ µm, and for the sample obtained by MSS, the value of the penetration depth is −9.607 × 10^−3^ µm.

**Figure 12:**
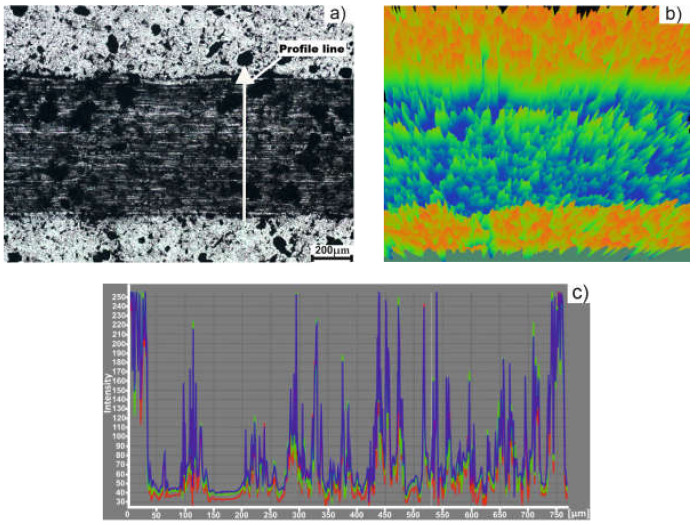
Sample obtained by TSS: (**a**) Worn track image (75X); (**b**) 3D image; (**c**) intensity profile.

In Figure 12, the worn track of the sample and the intensity profile are presented. As it can be seen from the intensity profile (Figure 12c), the width of the worn track is about 700 µm. Also, the profile shows the non-uniformity of the worn track, which is in accordance with the penetration depth. The non-uniformity can be also seen in the 3D image (Figure 12b).

**Figure 13:**
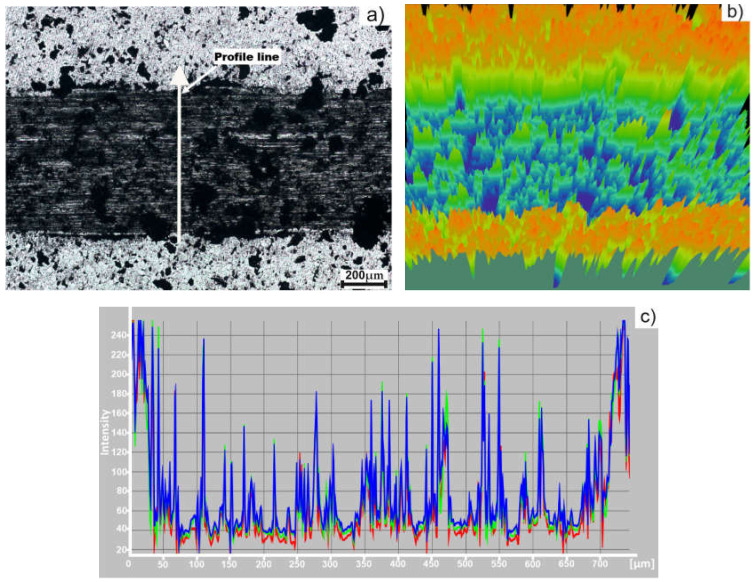
Sample obtained by MSS: (**a**) Worn track image (75X); (**b**) 3D image; (**c**) intensity profile.

Figure 13 presents the worn track and the intensity profile of the sample obtained by MSS. The width of the sample is approximatively 650 µm, as it can be seen from the intensity profile (Figure 13c).

**Figure 14:**
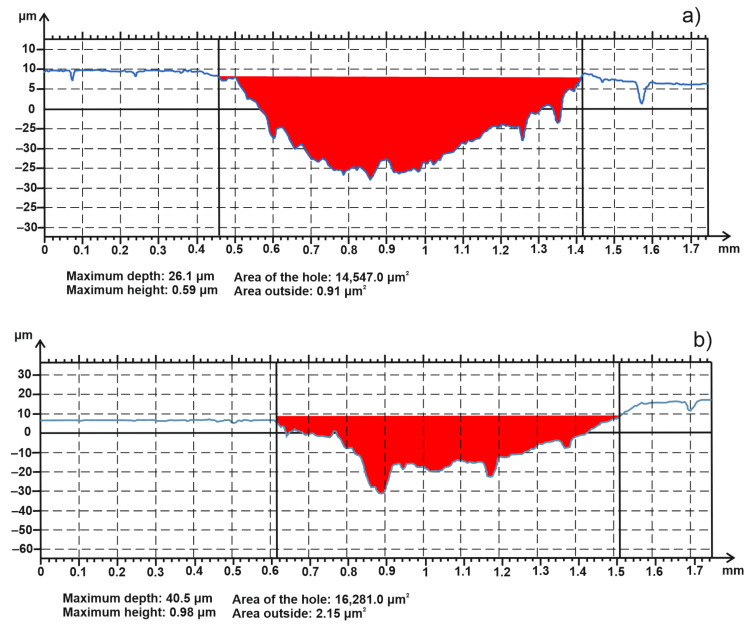
Worn track sections of the samples: obtained by: (**a**) TSS; (**b**) MSS.

In Figure 14, the worn track sections for both types of samples are presented.

Table 4 shows the values for worn track sections of the samples, the worn cap diameters of the balls, sample wear rates and partner wear rates.

From Figure 14 and Table 4, it can be noted that the wear rate of the sample obtained by MSS is higher compared to the other sample obtained by TSS. The same behavior can be observed for the friction coefficient, which is higher for the sample obtained by MSS than TSS. On the other hand, the MSS sample exhibited a lower partner wear rate than TSS. The lowest values of the partner wear rate confirm that the material of the sample adheres to the counter ball.

The samples obtained by the TSS process present better wear behavior in comparison with the ones obtained by MSS process due to the highest values of the densities of TSS samples leading to better mechanical properties. The average value of the wear coefficients is similar and fit within the values indicated in other studies dedicated to titanium-based alloy [22,48].

## 4. Conclusions

A titanium-based alloy with superior wear behaviour has been obtained using two sintering procedures. The structural analysis of the resulted sinters shows a relatively homogeneous distribution of the alloying elements, as well as the simultaneous presence of several crystalline phases. Two-step sintering or multiple-step sintering ensures lower energy consumption, which is a great advantage from an economic point of view compared to conventional sintering. The resulting alloy has a higher wear behavior than most titanium alloys. Friction coefficient values of 0.545 for the TSS samples, and 0.567 for those obtained by MSS, indicate a material with good antifriction properties. Due to its low weight, compared to the most used materials in the automotive area and its resistance to wear, the applicability of the as-obtained Ti-based alloy was configured in the field of automotive components, particularly the manufacturing of a connecting rod.

This research opens future works which will be focused on the decreasing of the particle size and to study the influence of different sintering treatments, such as spark plasma sintering (SPS) and microwave sintering (MWS) on the properties of Ti-based alloy. Also, Ti-based alloy as ultrafine powders can be used in the additive manufacturing (AM) process, in order to produce parts for automotive industry.

## Figures and Tables

**Table 1:** Data of the particle size distribution of the mixture.

d (nm)	G (d)	C (d)	d (nm)	G (d)	C (d)	d (nm)	G (d)	C (d)
441.0	0	0	1183.7	0	100	3177.1	0	100
482.4	0	0	1294.8	0	100	3475.5	0	100
527.7	0	0	1416.4	0	100	3801.8	0	100
577.3	0	0	1549.5	0	100	4158.9	0	100
631.5	6	4	1695.0	0	100	4549.4	0	100
690.8	20	18	1854.1	0	100	4976.6	0	100
755.7	100	88	2028.3	0	100	5444.0	0	100
826.6	15	98	2218.7	0	100	5955.2	0	100
904.3	2	100	2427.1	0	100	6514.5	0	100
989.2	0	100	2655.0	0	100	7126.3	0	100
1082.1	0	100	2901.4	0	100	7795.5	0	100

**Table 2:** Weight percentage of different elements identified in the investigated samples.

Sample Sintering Procedure	%Ti	%Al	%C	%Mn	%Zr	%Sn	%Others
TSS	87.1	6.5	2.4	3.0	0.7	-	0.3
MSS	86.1	6.1	2.5	4.0	0.8	-	0.5

**Table 3:** The values of the friction coefficients for both cases (TSS and MSS).

Sample Sintering Procedure	Friction Coefficient		
	Min	Max	Average Value
TSS	0.094	0.578	0.545
MSS	0.089	0.604	0.567

**Table 4:** The values for worn track sections and wear rates.

Sample Sintering Procedure	Worn Track Section (µm^2^)	Worn Cap Diameter (µm)	Sample Wear Rate (mm^3^·N^−1^·m^−1^)·10^−5^	Partner Wear Rate (mm^3^·N^−1^·m^−1^)·10^−5^
TSS	14547	589.5	182	4.129
MSS	16281	588.2	202.7	4.093

## Data Availability

Not applicable.
